# Fibroblast Activation Protein (FAP) Is Essential for the Migration of Bone Marrow Mesenchymal Stem Cells through RhoA Activation

**DOI:** 10.1371/journal.pone.0088772

**Published:** 2014-02-13

**Authors:** Kuei-Min Chung, Shu-Ching Hsu, Yue-Ru Chu, Mei-Yao Lin, Weir-Tong Jiaang, Ruey-Hwa Chen, Xin Chen

**Affiliations:** 1 Institute of Biotechnology and Pharmaceutical Research, National Health Research Institutes, Miaoli, Taiwan, Republic of China; 2 Vaccine Research and Development Center, National Health Research Institutes, Miaoli, Taiwan, Republic of China; 3 Institute of Biological Chemistry, Academia Sinica, Taipei, Taiwan, Republic of China; 4 Graduate Institute of Basic Medical Science, China Medical University, Taichung, Taiwan, Republic of China; National Institutes of Health, United States of America

## Abstract

**Background:**

The ability of human bone marrow mesenchymal stem cells (BM-MSCs) to migrate and localize specifically to injured tissues is central in developing therapeutic strategies for tissue repair and regeneration. Fibroblast activation protein (FAP) is a cell surface serine protease expressed at sites of tissue remodeling during embryonic development. It is also expressed in BM-MSCs, but not in normal tissues or cells. The function of FAP in BM-MSCs is not known.

**Principal Findings:**

We found that depletion of FAP proteins significantly inhibited the migration of BM-MSCs in a transwell chemotaxis assay. Such impaired migration ability of BM-MSCs could be rescued by re-expressing FAP in these cells. We then demonstrated that depletion of FAP activated intracellular RhoA GTPase. Consistently, inhibition of RhoA activity using a RhoA inhibitor rescued its migration ability. Inhibition of FAP activity with an FAP-specific inhibitor did not affect the activation of RhoA or the migration of BM-MSCs. Furthermore, the inflammatory cytokines interleukin-1beta (IL-1β) and transforming growth factor-beta (TGF-β) upregulated FAP expression, which coincided with better BM-MSC migration.

**Conclusions:**

Our results indicate FAP plays an important role in the migration of BM-MSCs through modulation of RhoA GTPase activity. The peptidase activity of FAP is not essential for such migration. Cytokines IL-1β and TGF-β upregulate the expression level of FAP and thus enhance BM-MSC migration.

## Introduction

Fibroblast activation protein (FAP) is a type II membrane serine protease with an extracellular catalytic domain. It belongs to the prolyl-cleaving peptidase family, which includes dipeptidyl peptidases 4 (DPP4), DPP8, DPP9, and DPP2. Unlike most cellular proteases, these enzymes preferentially cleave the peptide bond succeeding a proline residue in a polypeptide chain [1]. FAP is most homologous with DPP4, with 48% amino acid sequence identity. Unlike the ubiquitous expression pattern of DPP4, FAP is expressed exclusively in fetal cells, stromal fibroblasts, wounded tissues, and the stromal fibroblasts of more than 90% of malignant epithelial tumors, but not in benign tumors or normal adult tissues [2]–[4]. The forced expression of FAP in epithelial cells promoted cellular invasion through the extracellular matrix (ECM) and supported tumor growth in various xenograft animal models examined [5]–[8]. Recently, FAP showed immunosuppressive functions in a tumor microenvironment [9]. Depletion of FAP in stromal cells suppressed the growth of solid malignant tumors through interferon γ and tumor necrosis factor-alpha (TNF-α), although the underlying mechanism is not clear [9].

FAP is an endopeptidase with weak dipeptidase activity [10]. Its physiological substrate is α_2_-antiplasmin (α_2_-AP) [11], [12]. However, the biological implications of this function are not clear at present. FAP has type I collagen-specific collagenase activity *in vitro* [13], supporting the idea that FAP might be an ECM-degrading protease in the invasion and migration of tumor cells. When FAP is exogenously expressed in tumor cells, it is located in membranous protrusions at the leading edge of migrating tumor cells, interacting with β1 integrins and the cytoskeleton [14]–[16]. Overexpression of FAP in fibroblasts modifies the ECM to form a permissive microenvironment, promoting tumor invasion in human pancreatic cancers [17].

In normal adult tissues, FAP is only expressed in bone marrow mesenchymal stem cells (BM-MSCs) [18]. FAP can be used as a surface marker protein for the isolation of BM-MSCs from the bone marrow [18]. Moreover, FAP is also robustly expressed on freshly isolated murine multipotent bone marrow stromal cells [19]. Human MSCs are multipotent adult stem cells predominantly residing in the stromal compartment of hematopoietic bone marrow [20]. They are relatively easily isolated; they have immunosuppressive functions [21] and can differentiate to different tissue-specific cell types [22], [23]. Therefore, BM-MSCs have been explored intensively for potential clinical and therapeutic use with the aim of regenerating damaged tissues [24], [25]. What modulates the migration and homing of BM-MSC is thus an important research area attracting intense investigation in recent years, but the function of FAP in BM-MSCs is unknown. In this study, we investigated the roles of FAP in BM-MSCs. The effects of lentivirus-based short hairpin (sh)RNA in blocking FAP expression in BM-MSCs were examined.

## Materials and Methods

### Cell culture and virus infection

Detroit-551, IMR-90, and HFL-1 cell lines are purchased from American Type Culture Collection (ATCC). Human BM-MSCs were originally purified from bone marrow and grown as described [26]. They were cultured in fresh Iscove's Modified Dulbecco's Medium (IMDM, GIBCO) supplemented with 10% fetal bovine serum (FBS; Biological Industries), 1% l-glutamine (GIBCO), 0.1% penicillin–streptomycin (GIBCO), and 10 ng/ml basic fibroblast growth factor (bFGF) (Peprotech Inc.). Lentiviral systems for gene silencing were obtained from the Taiwan National RNAi Core Facility. Sequences of short hairpin RNA in the pLKO.1-puro vector are the following: pLKO.1-FAP shRNA-A (target sequence: 5′–CCCTCAGACAGTTTGCTTATT–3′) and pLKO.1-FAP shRNA-B (target sequence: 5′–GCATTGTCTTACGCCCTTCAA–3′). To generate recombinant lentiviruses, 293T cells were cotransfected with the packaging (pCMVΔ8.91), envelope (pMD.G), and shRNA-expressing (pLKO.1-puro) plasmids. The virus-containing supernatant was harvested to infect cells after determining the titers. To deplete FAP, cells were infected with lentiviruses expressing FAP shRNA at a multiplicity of infection (MOI) of 10 and incubated at 37°C overnight. Puromycin selection was carried out for 2 days by incubating the cells with fresh growth medium containing 2.5 µg/ml puromycin. The study protocols were approved by the Institutional Review Board of Human Subject Research Ethics Committee of Academia Sinica (AS-IRB01-10113).

### The expression construct of FAP revertant

Full-length FAP was generated by PCR from human cDNA libraries, and cloned to pLKO_ AS3w vector (Taiwan National RNAi Core Facility). Site-directed mutagenesis was carried out using the QuikChange kit (Strategen) to generate nucleotide changes at Leucine26 (from TTA to CTG) and Serine29 (from TCA to AGC) on FAP. Primers specific for each mutation were designed, and their sequences are as follows: Leucine26 (CTG), 5′–GATGTGCATTGCTCTGCGCCCTTCAAGAG–3′ (forward primer), 5′–CTCTTGAAGGGCGCAGGACAATGCACATC–3′ (reverse primer); Serine29 (AGC), 5′–TTGTCCTGCGCCCTAGCAGAGTTCATAACTC–3′ (forward primer), 5′–GAGTTATGAACTCTGCTAGGGCGCAGGACAA–3′ (reverse primer).

### Quantitative reverse transcription polymerase chain reaction (qRT–PCR)

qRT–PCR was carried out according to the published protocol [27]. RNA of each cell line was extracted by TRI REAGENT (Molecular Research Center Inc.) according to the manufacturer's instructions. RNA was converted to cDNA using ImProm-II Reverse Transcriptase (Promega) and qRT–PCR was performed using an ABI Prism 7900 Sequence Detection System with SYBR Green PCR Master Mix (Applied Biosystems). The sequences of primer used for qRT–PCR were: 5′–TGCTCTCTGGTGGTCTCCTAA–3′ (forward primer) and 5′–TTAGGAGACCACCAGAGAGCA–3′ (reverse primer) for FAP (GenBank Accession No. NM_004460.2); 5′–GAAGGTGAAGGTCGGAGTCA–3′ (forward primer) and 5′–TGGAAGATGGTGATGGGATT–3′ (reverse primer) for the gene for glyceraldehyde 3-phosphate dehydrogenase (GAPDH; GenBank Accession No. J04038.1). The threshold cycle (Ct) value for FAP was calculated against a normalization constant derived after correction against the control value for GAPDH as described [27].

### Immunoblotting analysis

To determine the protein level of FAP, cells were washed twice with phosphate-buffered saline (PBS) and lysed with NP-40 lysis buffer (10 mM HEPES pH 7.5, 142.5 mM KCl, 5 mM MgCl_2_, 1 mM EDTA, 0.2% NP-40, and a protease inhibitor cocktail from Roche). The supernatants were collected and the protein concentrations were determined using a Bradford assay. Total cell lysates (20 µg) were fractionated by sodium dodecyl sulfate–polyacrylamide gel (10%) electrophoresis (SDS–PAGE), transferred to nitrocellulose, and immunoblotted with an anti-FAP monoclonal antibody (Abnova), which is specific to FAP. After detection using a horseradish peroxidase (HRP)-conjugated secondary goat anti-mouse antibody (Jackson) and enhanced chemiluminescence (ECL) detection (Millipore), the quantification of expression level of FAP was manipulated by ImageJ software.

### Flow cytometry

The surface markers of BM-MSCs were analyzed by flow cytometry [26].The following antibodies against human antigens were used: phycoerythrin (PE)-labeled anti-CD34, anti-CD73, and anti-CD105, and fluorescein isothiocyanate (FITC)-labeled anti-CD90. All antibodies were purchased from BD Pharmingen except for anti-CD105, which was purchased from eBioscience. Surface-labeled cells were analyzed using a BD FACSCalibur flow cytometer (BD Bioscience). Results are expressed as percentages of positive cells.

### Chemokine and inflammatory antibody marker arrays

A lentiviral system obtained from Taiwan National RNAi Core Facility was used for gene silencing in BM-MSCs [28]. Lentivirus-infected cells (5×10^4^) were cultured in fresh growth medium (IMDM supplemented with 10% FBS, 1% l-glutamine, 0.1% penicillin–streptomycin, and 10 ng/ml bFGF) in a 6-cm tissue culture plate for 3 days. Culture supernatants were collected and assayed using the RayBio human chemokine antibody array 1 and the RayBio human inflammatory marker antibody array 3 (RayBiotech, Norcross, GA, USA). The assays were performed in accordance with the manufacturer's instructions.

### Transwell migration assay

A modified Boyden chamber assay was used to investigate the *in vitro* migration of BM-MSCs [29]. BM-MSCs (2×10^4^) or FAP shRNA-infected BM-MSCs were added to the upper wells of 8 µm pore size transwell chambers (BD Biosciences) in IMDM containing 1% l-glutamine in a 24-well tissue culture plate. Migration-inducing substances TNF-α (100 ng/ml), interleukin-6 (IL-6) (100 ng/ml) (PeproTech Inc.) or 10% FBS were added to the lower wells of the 24-well tissue culture plate in the same medium. The plate was incubated for 48 h at 37°C in a humidified atmosphere, and migration of BM-MSCs across the membrane was subsequently determined by counting the cell numbers in both upper and lower chambers. For migratory inhibition experiments, BM-MSCs were incubated with a synthetic potent FAP inhibitor, 2F09 (10 µM) (compound 19 in the paper reported by Tsai et al., 2010) [30] or 2F01 (10 µM) (compound 2 in a recent publication) [31] before being transferred into the upper chamber. The inhibitor was added to the medium in both upper and lower chambers at the same concentration.

### Expression and purification of glutathione S-transferase (GST) fusion proteins

Overexpression and purification of GST fusion proteins were performed as described [32]. *Escherichia coli* BL21 was transformed with plasmids expressing the GST–Rhotekin Rho binding domain (GST–RBD) or GST-tagged human PAK1–p21 binding domain (PAK1–PBD), inoculated into LB medium supplemented with 100 µg/ml ampicillin and grown at 37°C. After induction with IPTG at 0.2 mM at 25°C overnight, bacteria were harvested by centrifugation and lysed in lysis buffer (50 mM Tris-HCl pH 7.5, 150 mM NaCl, 5 mM MgCl_2_, 1% Triton X-100, 1 mM DTT, and Roche protease inhibitor cocktail) containing 1 mg/ml lysozyme. After incubation at 4°C for 1 h, the cell lysates were sonicated. The supernatant was collected and mixed with glutathione–sepharose 4B beads (GE Healthcare) at 4°C for 2 h. The beads were washed ten times using lysis buffer and resuspended in the same buffer containing 10% glycerol.

### Assay for GTP-bound Rho GTPase

Immunoprecipitations of GTP-bound Rho GTPase using cell lysates containing equal amounts of proteins and purified GST fusion proteins bound to the glutathione–sepharose beads were performed as described [32]. Cells in a 6-cm culture dish were washed with TBS buffer (10 mM Tris-HCl pH 8.0, 150 mM NaCl) and lysed using lysis buffer (50 mM Tris-HCl pH 7.5, 200 mM NaCl, 10 mM MgCl_2_, 1% Triton X-100, 1 mM DTT, 5% glycerol, and Roche protease inhibitor cocktail). The cell debris was removed by centrifugation at 20,000 g for 20 min at 4°C and supernatants were collected. Protein concentrations were determined using Bradford assays. Equal amounts of cell extracts were loaded for immunoblotting. The same cell extract of 1 mg was diluted to 1 ml with binding buffer (25 mM Tris-HCl pH 7.5, 40 mM NaCl, 30 mM MgCl_2_, 0.5% Triton X-100, 1 mM DTT, and Roche protease inhibitor cocktail) and incubated with 20 µg of purified GST–RBD or GST-tagged PAK1–PBD beads at 4°C with gentle rotation for 1 h. The pull-down complexes were washed with binding buffer five times, and subjected to immunoblotting to detect the GTP-bound Rho GTPase.

### Statistical analysis

Means and standard deviations were calculated using Microsoft excel. The statistically significant differences between the groups were assessed using a two-tailed Student's t test. The level of significance was set at p<0.05.

## Results

### FAP expressed in culture-expanded BM-MSCs

**Figure 1:**
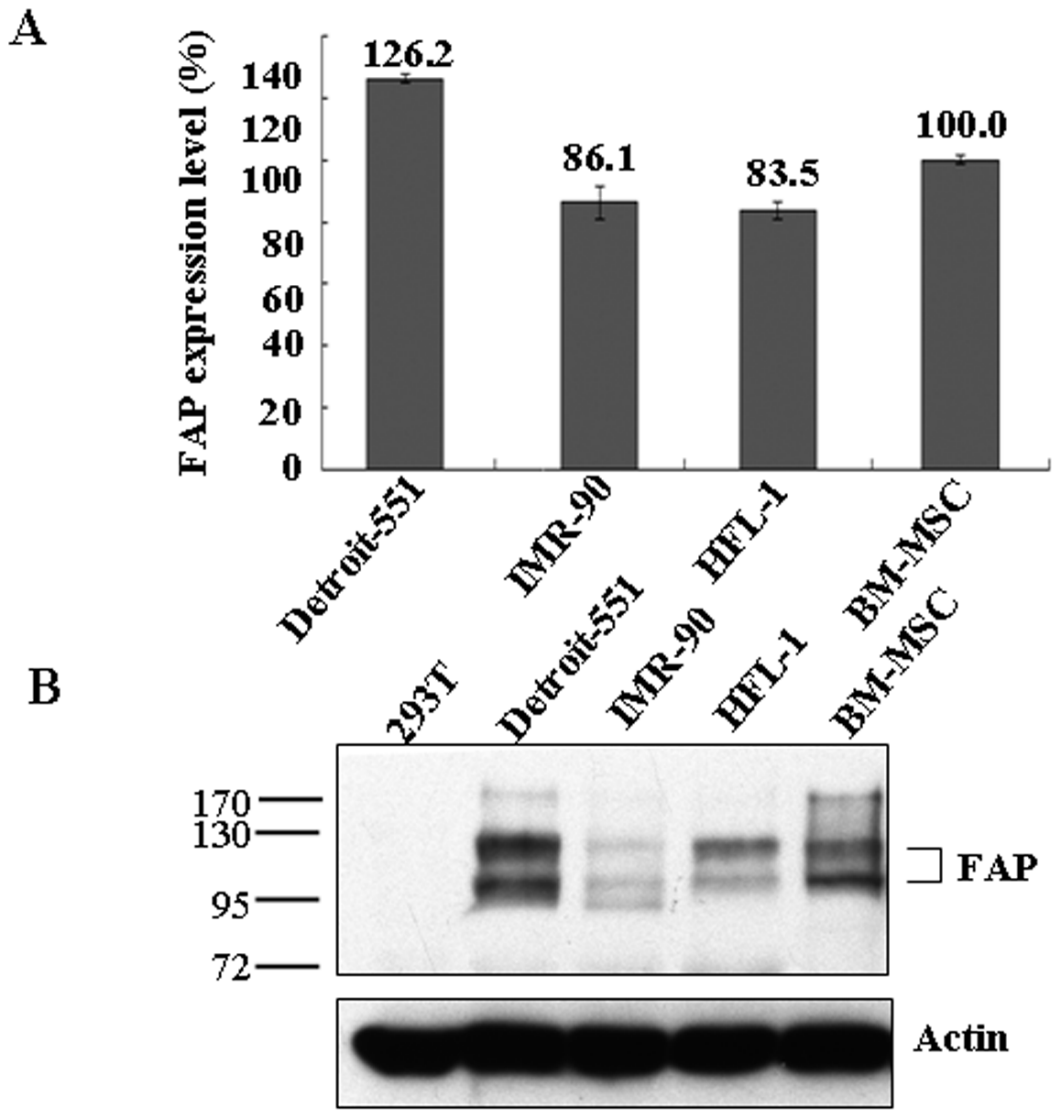
Characterization of FAP expression level in BM-MSCs. (A) Analysis of the mRNA expression levels of FAP in different human cell lines as indicated. Quantification was carried out by qRT–PCR as described in the Materials and methods section. The error bars are showing standard deviation (SD). (B) Expression of FAP detected by western blot analysis with anti-FAP antibodies. Immunoblotting with anti-actin antibodies was used as a loading control.

FAP is expressed in fetal fibroblasts and BM-MSCs [2], [3], [33], [34]. First, we compared its mRNA expression level in BM-MSCs with those of fetal fibroblasts by qRT–PCR. We found that the mRNA expression level of FAP in BM-MSCs was similar to those in human fetal fibroblasts including Detroit-551, IMR-90, and HFL-1 cell lines (Fig. 1A). Next, we confirmed this finding by examining the protein level of FAP in these cell lines by immunoblotting. The FAP proteins were detected at similar levels in the cell lysates of BM-MSCs and fetal fibroblasts (Fig. 1B). For comparison, there was little FAP protein in 293T cells, an epithelial type of cancer cell (Fig. 1B). Thus, FAP was expressed in our culture-expanded BM-MSCs, so these could be used for further studies as presented below.

### FAP depletion in BM-MSCs by lentiviral infection

**Figure 2:**
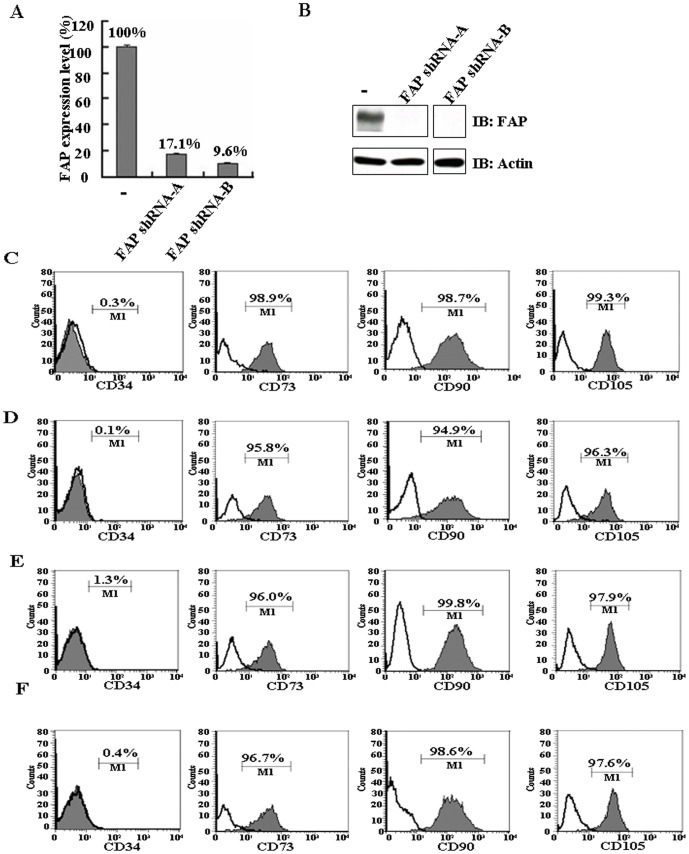
FAP depletion in BM-MSCs by lentiviral infection. (A) BM-MSCs were depleted of FAP by transfection with lentiviruses encoding two FAP shRNA sequences. The efficiency of shRNA-based downregulation was determined by qRT–PCR. Results are given as the percentage of change in mRNA expression relative to pLKO empty vector-infected cells set as 100%. The error bars are showing standard deviation (SD). (B) The efficiency of shRNA-based downregulation was determined by western blot analysis. Data are representative of three independent experiments. (C–F) Flow cytometric analysis of cell surface markers on FAP-depleted BM-MSCs. (C) Parental BM-MSCs were analyzed using antibodies against CD34, CD73, CD90, and CD105. (D) Vector infected BM-MSCs were analyzed using antibodies against CD34, CD73, CD90, and CD105. (E) As in (D) but with FAP shRNA-A infected BM-MSCs. (F) As in (D) but with FAP shRNA-B infected BM-MSCs.

To characterize the cellular function of FAP in BM-MSCs, we investigated the effect of FAP on cellular function by depleting FAP using lentiviruses coding for FAP shRNA. The target sequence of shRNA-A is in the 3′ untranslated region (3′UTR) of FAP, while shRNA-B is in the coding region. Both FAP shRNA-A and FAP shRNA-B drastically decreased the mRNA expression level of endogenous FAP to 17.1% and 9.6% of control levels, respectively (Fig. 2A). Consistent with the qRT–PCR data, the endogenous FAP proteins could not be detected in the cell lysates of BM-MSCs after such depletion of FAP expression (Fig. 2B). Thus, the two shRNAs could efficiently deplete FAP.

Before using these shRNAs for further studies, we carefully compared the expression of the cell surface markers characteristic of BM-MSCs. Flow cytometry data showed that parental BM-MSCs were negative for the hematopoietic cell marker, CD34, and positive for the BM-MSC-related surface markers, CD73, CD90, and CD105 (Fig. 2C). The profile of the markers was not changed when using FAP-depleted BM-MSCs (Fig. 2D–F), indicating that FAP depletion did not alter the cellular markers of BM-MSCs.

### FAP is involved in the migration of BM-MSCs

**Figure 3:**
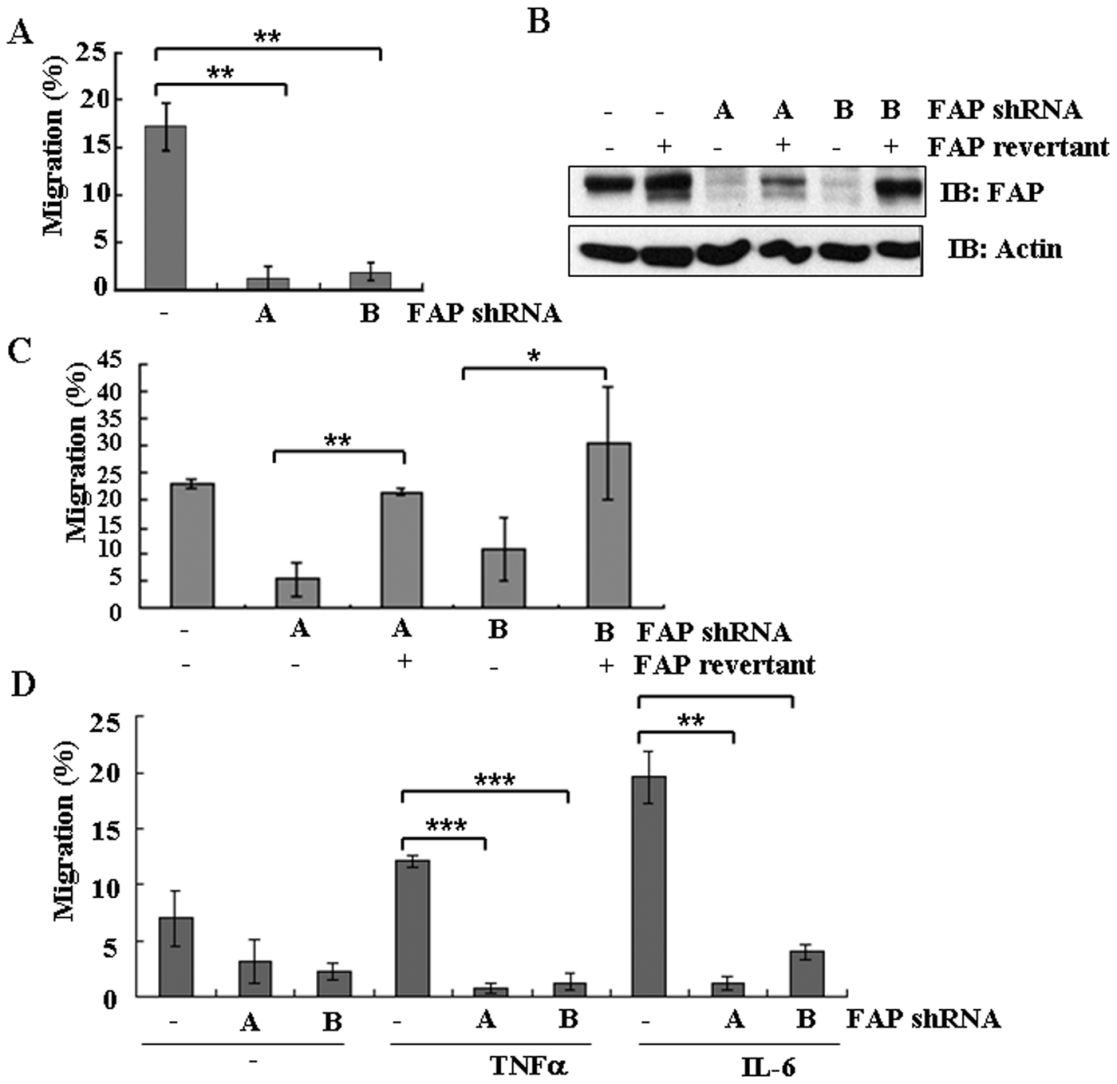
FAP depletion and the migration of BM-MSCs. (A) BM-MSCs were infected by lentiviruses encoding FAP shRNA-A (A), shRNA-B (B), or vector control (–). The migration ability of FAP-depleted BM-MSCs in the presence of 10% FBS was analyzed by Boyden chamber transwell assays *in vitro*. Cells were seeded in the upper wells of the chambers in IMDM and the lower wells contained the same medium with 10% FBS. Cells were allowed to migrate for 48 h and the numbers of cells that migrated were determined as described in the Materials and methods section. Data are representative of three separate experiments each performed in triplicate. The error bars are showing standard deviation (SD). (B) FAP-depleted BM-MSCs were infected with lentiviruses expressing an FAP revertant. The protein expression level of FAP revertant in FAP knockdown BM-MSCs was determined by western blot analysis. (C) Results are shown as in (A) but with the FAP-depleted cells infected with a lentivirus expressing an FAP revertant. Data are representative of three separate experiments each performed in triplicate. The error bars are showing standard deviation (SD). (D) Results are shown as in (A) but the lower wells of the transwell chambers contained IMDM with TNF-α (100 ng/ml) or IL-6 (100 ng/ml). Data are representative of three separate experiments each performed in triplicate. Means and standard deviations are given in each case. The error bars are showing standard deviation (SD). The statistically significant differences between the groups were assessed using a two-tailed Student's t test. The degree of significance is indicated as fellows: *p<0.05; **p<0.001; ***p<0.0001.

We aimed to determine whether FAP might play a role in the migration of BM-MSCs. The transwell migration assays were performed in the presence of 10% FBS, which measures spontaneous migration. We found that depletion of human FAP by both shRNAs significantly inhibited the migration of BM-MSCs compared with vector-infected control (Fig. 3A). The migration of BM-MSCs decreased from 17.2% to less than 1.1% and 1.7% for FAP shRNA-A and FAP shRNA-B, respectively. To determine whether the defect is specific to FAP depletion, an FAP revertant was constructed to re-express FAP in the FAP-depleted cells. The FAP revertant encodes the wild-type FAP protein with nucleotide changes at Leucine26 (from TTA to CTG) and Serine29 (from TCA to AGC), sequences that are no longer targets for the two shRNAs used in this study. Expression of the revertant restored the expression level of FAP in these FAP-depleted BM-MSCs, as confirmed by immunoblotting (Fig. 3B). Importantly, the ability of BM-MSCs to migrate was rescued by this revertant (Fig. 3C), indicating that FAP is responsible for the phenotype we observed. Thus, FAP plays an essential role in the migration of BM-MSCs.

### Effects of FAP on chemokine-stimulated migration of BM-MSCs

TNF-α and IL-6 are reported to stimulate BM-MSC migration [35], [36]. When added to the serum-free medium in the chambers, both chemokines stimulated the migration of BM-MSCs as reported [35], [36], from 7.0% to 12.1% and 19.6%, respectively (Fig. 3D). Next, we tested whether the stimulatory function of these two chemokines required the presence of FAP. Interestingly, when FAP was depleted, the BM-MSCs failed to migrate in the presence of TNF-α or IL-6 (Fig. 3D). Thus, FAP plays an essential role in BM-MSC migration stimulated by TNF-α and IL-6.

### Effect of FAP depletion on the activation of RhoA GTPase

**Figure 4:**
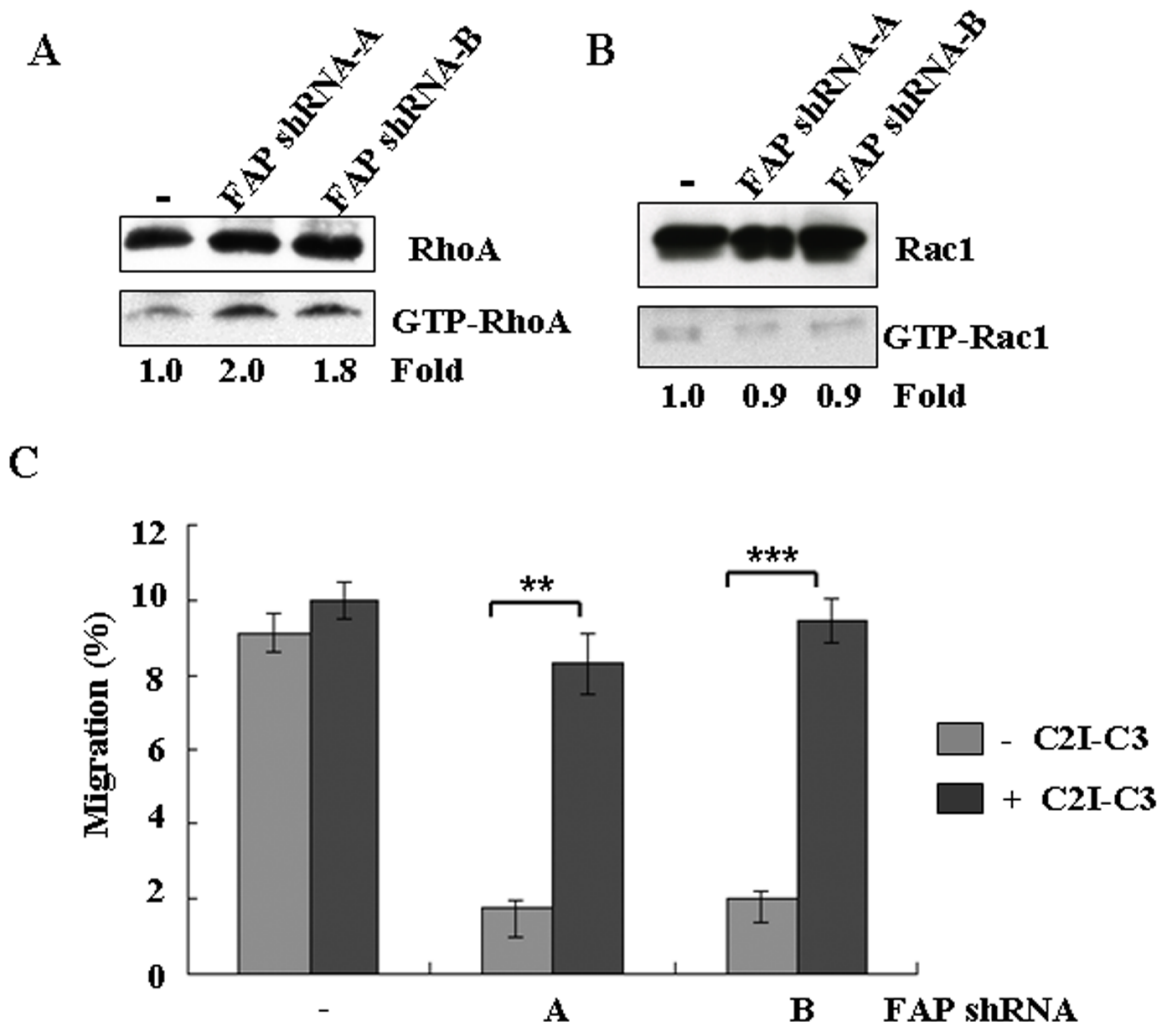
Rho GTPase activation in FAP-depleted BM-MSCs. (A) The amount of GTP-bound RhoA was determined with the cells depleted of FAP, as described in the Materials and methods section. The amounts of GTP-bound RhoA were normalized against total RhoA protein present in cell lysates and expressed as fold induction compared with the cells infected with the vector control. Data are representative of two independent experiments. (B) Results are shown as in (A) except that GTP-bound Rac1 was used. Data are representative of two independent experiments. (C) Effect of RhoA inhibitors on the migration of FAP-depleted BM-MSCs was determined by transwell assays. FAP-depleted BM-MSCs were pretreated without or with the RhoA inhibitor C2I-C3 (1 µg/ml) (Cytoskeleton Inc.) in IMDM for 2 h and seeded in the upper wells of the chambers. The lower wells contained IMDM with 10% FBS. Cells were allowed to migrate for 48 h and absolute cell numbers were determined as described in the Materials and methods section. Data are representative of three separate experiments each performed in triplicate. Means and standard deviations were calculated. The error bars are showing standard deviation (SD). The statistically significant differences between the groups were assessed using a two-tailed Student's t test. The degree of significance is indicated as fellows: **p<0.001; ***p<0.0001.

Rho GTPases regulate cell migration in BM-MSCs [37], and the activation of Rho GTPase is positively correlated with the level of GTP-bound RhoA [32], [38]. When RhoA activity is inhibited, the migration of BM-MSCs is enhanced when subjected to exogenous stimulants [37]. We tested whether the activities of the Rho family of GTPases would be affected when FAP was depleted. Depletion of human FAP by shRNA resulted in almost a 2-fold elevation in RhoA activity, as determined by the amount of GTP-bound RhoA (Fig. 4A). In contrast, Rac1 activity was not changed when FAP was depleted (Fig. 4B). It is possible that FAP depletion led to the activation of RhoA, leading to inhibition of the migration of BM-MSCs. To test this, we treated the FAP-depleted BM-MSCs with the specific RhoA inhibitor C2I-C3, and evaluated its effect on cell migration. RhoA inhibition did not affect the migration of vector-infected BM-MSCs (Fig. 4C). However, the migration of FAP-depleted BM-MSCs treated with the two shRNA constructs was largely restored after treatment with this inhibitor (Fig. 4C). Our results indicate that FAP modulates the migration of BM-MSCs via the activity of RhoA; thus, depletion of FAP activated RhoA GTPase and inhibited the migration of BM-MSCs.

### FAP peptidase activity is not essential for the migration of BM-MSCs

**Figure 5:**
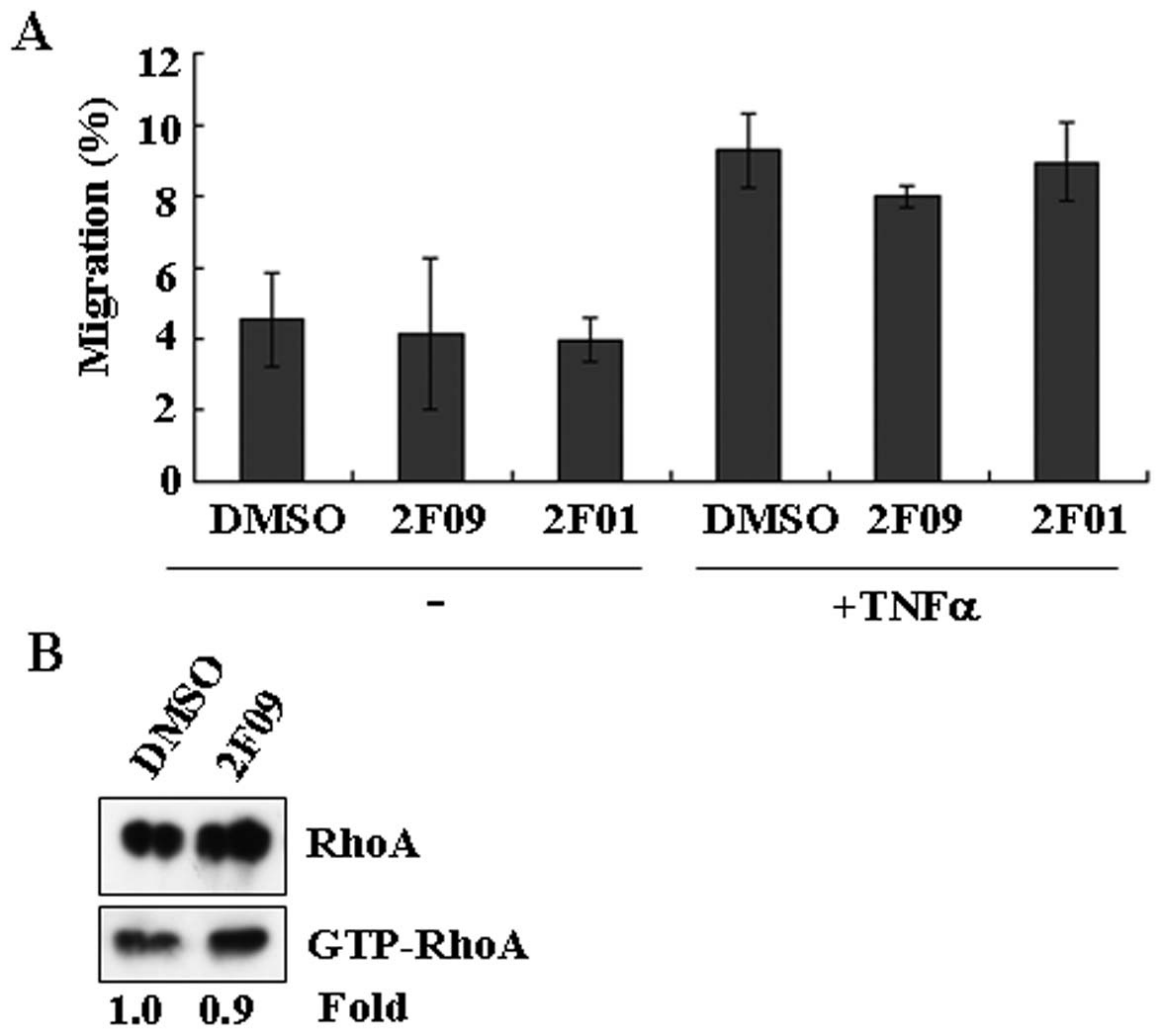
Inhibition of FAP activity had no effect on BM-MSC migration. (A) BM-MSCs were incubated with DMSO, 2F09 (10 µM) or 2F01 (10 µM) in IMDM before being seeded in the upper wells of transwell chambers. The lower wells contained IMDM with TNF-α (100 ng/ml). Cells were allowed to migrate for 48 h and absolute cell numbers were determined as described in the Materials and methods section. Data are representative of three separate experiments each performed in triplicate. The error bars are showing standard deviation (SD). (B) Results are shown as in (A) except that the cells were incubated with dimethyl sulfoxide (DMSO) solvent or 2F09 (10 µM) in IMDM for 2 h before lysis to determine the amount of GTP-bound RhoA as described in the Materials and methods section. Data are representative of two independent experiments.

To understand whether the peptidase activity of FAP is essential in the migration of BM-MSCs, 2F09, a potent FAP inhibitor, was used to inhibit the enzyme activity of FAP. This is a potent and selective chemical inhibitor of FAP, with an IC_50_ of 22 nM (compound 19 in the paper reported by us) [30]. Migration assay was performed under the same experimental condition as described in Figure 3D, except in the presence of 10 µM 2F09. This treatment had no effect on migration compared with the controls (Fig. 5A). To eliminate the possibility of the compound side effect, we have tested with another FAP inhibitor, 2F01, compound 2 in a recent publication [31], which has a different chemical structure from 2F09 [30], [31]. Inhibition of FAP activity with 2F01 also did not affect the migration of BM-MSCs (Fig. 5A). Because these two compounds are very potent inhibitors for FAP enzymatic activities with very different pharmacophores, the results support that the enzymatic activity of FAP is not required for the migration of BM-MSCs.

Next, we tested whether the activity of RhoA was changed after treatment with 2F09. BM-MSCs were treated with 10 µM 2F09 and the activation of Rho GTPase was monitored by detecting GTP-bound RhoA. There was no difference in RhoA GTPase activity with or without the treatment with 2F09 (Fig. 5B). Thus, inactivation of FAP by 2F09 did not alter the activation status of RhoA GTPase or the migration of BM-MSCs, so the enzymatic activity of FAP is not required for the migration of BM-MSCs.

### Depletion of FAP and the protein levels of inflammatory cytokines and chemokines

**Figure 6:**
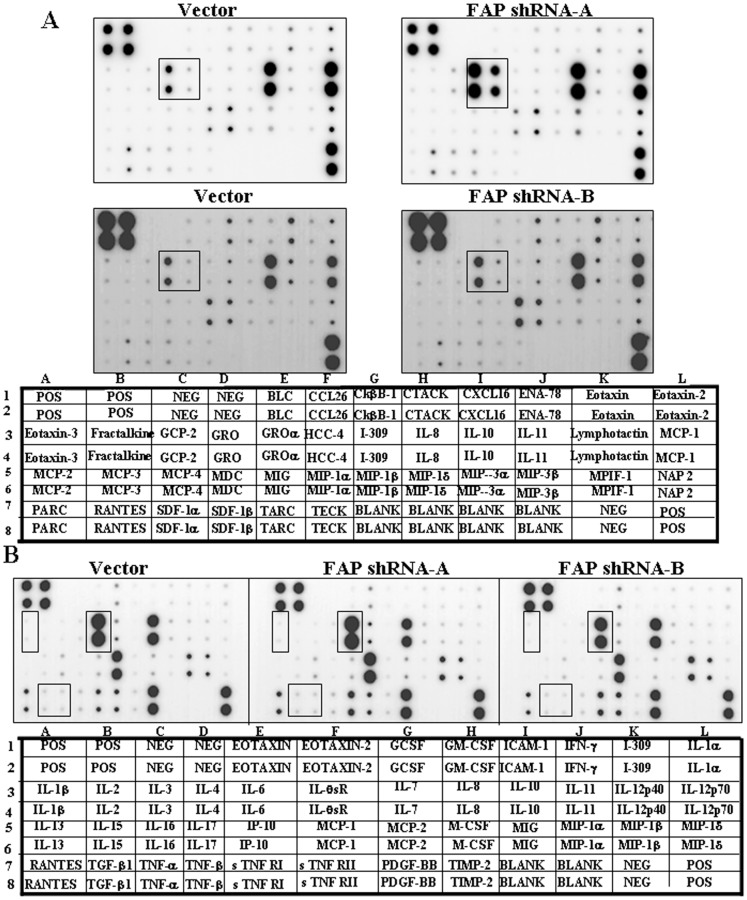
Effect of FAP depletion on chemokine and inflammatory cytokine secretion profiles. (A) The protein levels of chemokines in FAP-depleted BM-MSCs were compared to the control with RayBio human chemokine antibody array 1, according to the manufacturer's manual. The corresponding chemokines spotted on the membrane was shown below the blot. The highlighted box in the blot indicated the position of GRO and GRO-α. Data are representative of two independent experiments. (B) As in (A) but with RayBio human inflammation antibody array 3. The highlighted box in the blot indicated the position of cytokines, IL-1β, IL-6, TGF-β and TNF-α, which are known to involve in cellular movement. Representative results of two independent experiments.

Inflammatory cytokines and chemokines play critical roles in regulating trafficking of BM-MSCs [35], [36], [39]. We compared the protein levels of secreted cytokines and chemokines using chemokine protein membrane arrays before and after FAP depletion. Compared with the vector-infected control, the chemokine growth-regulated oncogene (GRO) level was elevated, while the levels of other chemokines were not changed significantly (Fig. 6A). We ruled out the possibility that GRO was a substrate of FAP because we did not detect any cleavage of recombinant GRO by purified FAP *in vitro* (data not shown). We found that the elevated protein level of GRO was caused by an increased mRNA expression level, as determined by microarray and qRT–PCR assays (data not shown). In addition, we also confirmed that GRO did not have any effect on the migration of BM-MSCs (data not shown). Thus, the effect of FAP depletion on the migration of BM-MSCs is most likely not directly a result of the elevation of GRO expression. The cause of the increase in GRO expression upon FAP depletion remains unclear.

Inflammatory cytokine array assays were also used to compare control cells and the two lines of FAP-depleted BM-MSCs. We did not detect any changes after FAP depletion on L-1β, TGF-β, TNF-α, or IL-6, which are known to be involved in cellular movement (Fig. 6B). Thus, depletion of FAP did not affect the expression levels of these chemokine and inflammatory cytokines in BM-MSCs.

### Cytokines IL-1β and TGF-β modulate the expression level of FAP in BM-MSCs

**Figure 7:**
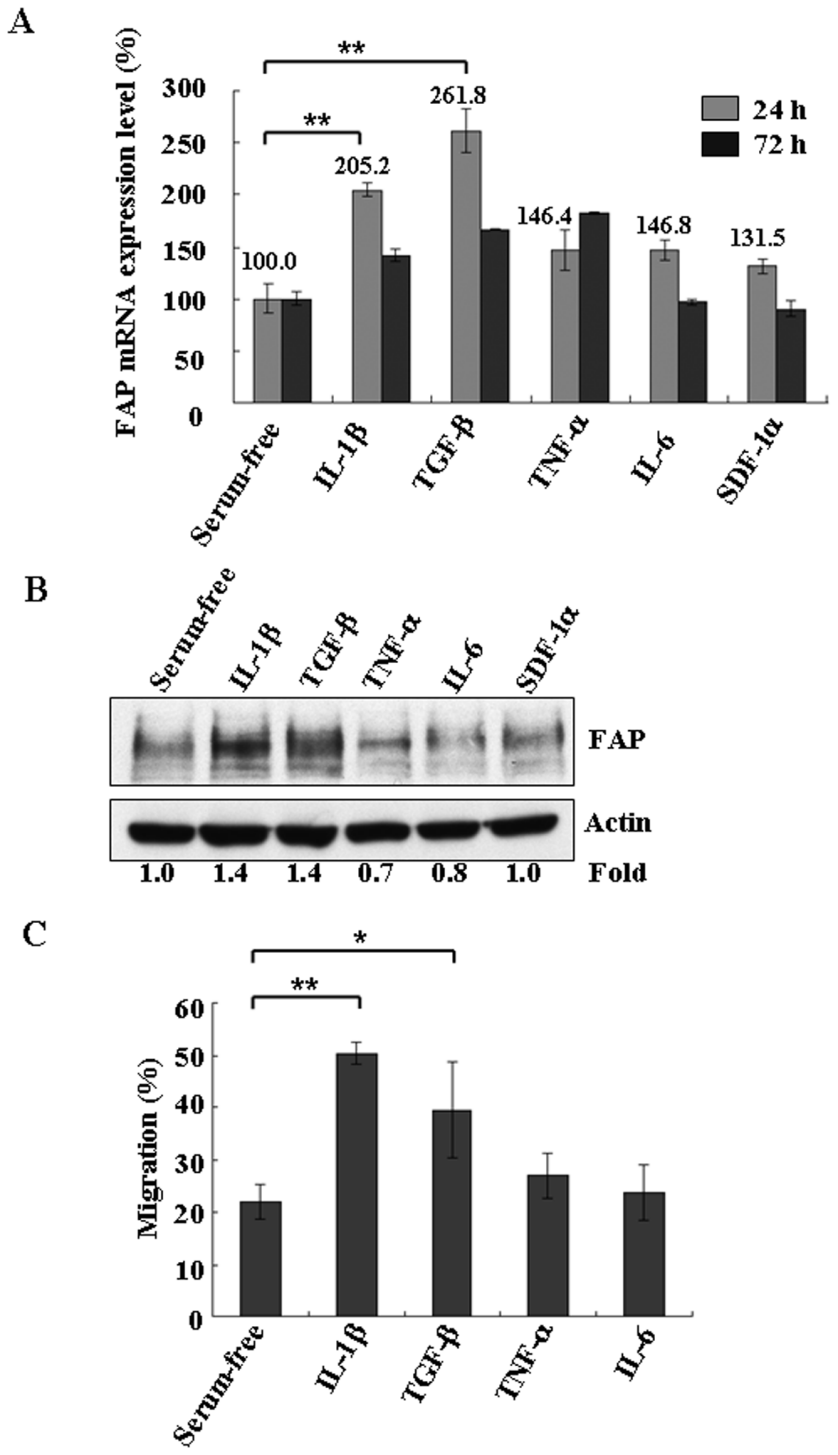
IL-1β and TGF-β increased the expression of FAP in BM-MSCs. (A) BM-MSCs were incubated with IL-1β (100 ng/ml), TGF-β (100 ng/ml), TNF-α (100 ng/ml), IL-6 (100 ng/ml), or SDF-1α (100 ng/ml) under serum-free conditions in IMDM for 24 or 72 h. The cells were harvested and lysed to determine the mRNA levels of FAP by qRT–PCR. Results are given as the percentage change in mRNA expression relative to untreated cells, which was set at 100%. Data are representative of three independent experiments. The error bars are showing standard deviation (SD). (B) Results are as shown in (A) except that the protein expression level of FAP was determined after incubation for 48 h with the indicated cytokines/chemokines in IMDM. Immunoblotting was carried out with anti-FAP antibodies. Immunoblotting for actin was used as a loading control. Data are representative of three independent experiments. (C) The effects of IL-1β and TGF-β on the migration of BM-MSCs were determined by transwell assays. BM-MSCs were incubated without or with IL-1β (100 ng/ml), TGF-β (100 ng/ml), TNF-α (100 ng/ml) and IL-6 (100 ng/ml) grown under serum-free conditions in IMDM for 48 h before being seeded in the upper wells of transwell chambers. The lower wells contained TNF-α (100 ng/ml) in IMDM to stimulate migration. Cells were allowed to migrate for 24 h and absolute cell numbers were determined as described in the Materials and methods section. Data are representative of three separate experiments each performed in triplicate. Means and standard deviations were calculated. The error bars are showing standard deviation (SD). The statistically significant differences between the groups were assessed using a two-tailed Student's t test. The degree of significance is indicated as fellows: *p<0.05; **p<0.001.

Inflammatory cytokines such as IL-1β, TGF-β, and TNF-α are found to be increased in damaged and inflamed tissues [40]. To determine the effect of these cytokines on FAP expression, we treated the cells with inflammatory cytokines for 24 and 72 h. Incubation with IL-1β (100 ng/ml) or TGF-β (100 ng/ml) for 24 h increased the mRNA expression of FAP about 2.0- and 2.6-fold, respectively, when compared with untreated control cells (Fig. 7A). In comparison, the mRNA level of FAP increased only slightly after 24 h of incubation with TNF-α (100 ng/ml), IL-6 (100 ng/ml), or stromal cell-derived factor-1alpha (SDF-1α)(100 ng/ml) (Fig. 7A). After 72 h incubation, the FAP level was largely back to baseline (Fig. 7A). Consistent with the mRNA data (Fig. 7A), the protein level of FAP was increased after treatment with IL-1β and TGF-β for 24 h compared with the untreated control (Fig. 7B). The results indicate that IL-1β and TGF-β can regulate the expression of FAP.

In the *in vitro* migration assay, the migration of MSCs was not stimulated by IL-1β or TGF-β (data not shown), similar to what was reported before [41], [42]. Because these two cytokines increased the protein level of FAP (Fig. 7B), we asked whether the TNF-α-stimulated migration will be affected when we pre-treated the cells first with IL-1β or TGF-β. When the cells were pretreated with these two inflammatory cytokines in serum-free medium for 48 h followed by treatment with TNF-α to stimulate migration, we found that pre-treatment of these two chemokines stimulated the TNF-α-stimulated migration of BM-MSCs by 2.3- and 1.8-fold, respectively (Fig. 7C). The migration of BM-MSCs increased only slightly after pre-treatment with TNF-α (100 ng/ml) or IL-6 (100 ng/ml) (Fig. 7C) indicating that pre-treatment of TNF-α or IL-6 has no additional effect on TNFα-stimulated migration. Because both IL-1β and TGF-β increased FAP expression (Fig. 7A and 7B) and also enhanced TNF-α-stimulated cell migration, there is a possible link between the amount of FAP and the migration ability of BM-MSCs as measured by stimulation with TNF-α.

## Discussion

Bone marrow-derived MSCs contribute to damaged tissue repair and regeneration, and recruitment of BM-MSCs to the target site is a complex multistep process [43]. The release of BM-MSCs from the bone marrow niche into the bloodstream is stimulated after sensing the signal from the injured tissue [43]. The signals might include cytokines or chemokines, their receptors, and proteolytic enzymes, though the mechanisms are not known in detail [44]. Several cytokines, including TNF-α, IL-6, and MCP-1, enhance the migration of MSCs *in vitro* (Fig. 3D) [35], [36], [39]. Several inflammatory cytokines, including TGF-β, IL-1β and TNF-α upregulated the expression of matrix metalloproteinases (MMPs) in BM-MSCs, resulting in enhanced migration through the ECM [45]. Here, we found that the prolyl-cleaving peptidase FAP was required for the migration of BM-MSCs (Fig. 3). FAP did not appear to regulate the expression or the cleavage of the chemokines or inflammatory cytokines investigated (Fig. 6). In fact, the peptidase activity of FAP was found not to be essential for the migration of BM-MSCs (Fig. 5). Instead, FAP contributed to the migration of BM-MSCs through modulating RhoA GTPase activity (Fig. 4).

The Rho family of GTPases regulates the organization and dynamics of actin cytoskeleton and cell migration and adhesion [46], [47]. Upon binding to GTP, these GTPases interact with and activate downstream effectors to exert their functions [38]. Interestingly, the effect of Rho activation on cell migration depends on the cell type. In cells that have stress fibers and focal adhesions, such as fibroblasts, inhibition of RhoA promotes migration by reducing these features [48]. In less strongly adherent cells, such as macrophages, neutrophils, and cancer cell lines, RhoA activation does not affect cell adhesion but is required for cell migration by inducing cell body contraction [49]-[51]. Rho signaling in stem cells has also been studied [37], [52]. Activation of the physiologically linked Rac GTPase was proposed to have a role in suppressing the proliferation and migration of hematopoietic stem and progenitor cells [52]. In BM-MSCs, RhoA activity was suppressed under normal growth conditions [37]. Activation of RhoA increased actin stress and inhibited the migration of BM-MSCs induced by physiological stimuli [37]. We confirm here that RhoA activation was inversely linked with the migration of BM-MSCs (Fig. 4). Moreover, the presence of FAP affected the activation status of RhoA in BM-MSCs (Fig. 4). When FAP was depleted, RhoA activation was increased (Fig. 4) so the migration of BM-MSCs was inhibited (Fig. 3). Consistent with the notion that RhoA activation is the cause of defective migration, addition of the RhoA inhibitor C2I-C3 reversed this suppression of migration when cells were depleted of FAP (Fig. 4C).

In the past, the activity of FAP has been the focus of several studies by investigating its potential cellular substrates [11], [13]. A function of FAP independent of its proteolytic activity has also been observed in tumor cells after forced expression. In those studies, both wild-type and proteolytically inactive mutants of FAP could promote cancer cell growth [53], [54]. Interestingly, the activity of FAP was not important in the migration ability of MSCs (Fig. 5). Instead, FAP appears to function as an important regulatory receptor or adaptor, instead of a proteolytic enzyme. It is not clear whether this non-proteolytic function of FAP observed is related to cell types. Moreover, it is not clear whether regulation of FAP activity and expression correlate with various cell types and/or physiological states.

It has been reported that Rho and Rac have an antagonistic relationship in which Rac1 can inactivate RhoA in some cell types [55], [56]. Rac1 activation can lead to Rho inactivation [56]. However, there is also evidence that RhoA activation can lead to Rac1 activation [57]. Nevertheless, we did not detect any change in Rac status upon RhoA activation in BM-MSCs (Fig. 4A, B). This might have arisen from a cell-type effect, or possibly any change in Rac1 activity was too small to be detected in this assay.

How does FAP regulate BM-MSC migration via the Rho family of enzymes? One possibility is through potential interactions of FAP with other signaling complexes on the plasma membrane [58]. Take the homologous protein of FAP, DPP4, as an example. Even though DPP4 only contains six amino acids in its cytoplasmic side, this short stretch of polypeptide interacts with CARMA-1, which is critical for its function of T-cell activation [59]. A similar mode of action might be adopted by FAP in its interaction with other signaling molecules. Supporting this, FAP has been found to associate with integrin α3β1 [14]. Integrin signaling is known to regulate the activation of Rho GTPases [60]. Suppression of RhoA-GTP levels is linked with activation of integrin α3β1 during HT-29 cell differentiation [61]. Putting these together, it is possible that the association of FAP with integrin modulates downstream RhoA activity, thus affecting the migration ability of BM-MSCs. More studies are required to investigate the functional partner(s) of FAP in the modulation of RhoA activity in BM-MSCs.

Inflammatory cytokines such as IL-1β, TGF-β, and TNF-α are present in damaged and inflamed tissues [40]. These cytokines are known to increase gelatinase gene expression in diverse cell types [62]-[65], and act as chemoattractants to increase the invasive capacity of human MSCs by upregulation of the activities of several enzymes including MMP-2, MT1-MMP, and MMP-9 [45]. The inflammatory cytokines IL-1β and TGF-β are reported to induce FAP expression in the highly metastatic ovarian cancer cell line HO-8910PM [66]. Interestingly, the treatment of the BM-MSCs with TGF-β and IL-1β also increased the expression of FAP and the migration of BM-MSCs stimulated by TNF-α (Fig. 7). Because the migration of BM-MSCs requires FAP (Fig. 3), we hypothesize that the amount of FAP is positively linked with the migration ability of BM-MSCs. We measured the migration ability of BM-MSCs by stimulation with TNF-α, which does not affect the expression level of FAP significantly (Fig. 7). The stimulation of migration by TNF-α is dependent on FAP (Fig. 3). Pre-treatment of BM-MSCs with TGF-β and IL-1β increase expression of FAP, and also enhanced TNF-α-stimulated cell migration(Fig. 7). On a broader sense, the presence of these cytokines upon tissue damage might increase the expression of FAP in BM-MSCs, thus enhancing the migration of BM-MSCs to damaged tissue sites. Whether this is the case awaits further studies.

In conclusion, FAP is essential for the migration of BM-MSCs through modulation of RhoA GTPase activity. However, peptidase activity of FAP does not play a role in migration. FAP does not directly regulate or process the expression of the chemokine and inflammatory cytokines, which are known to be involved in cellular movement. Instead, we found that the inflammatory cytokines IL-1β and TGF-β promoted BM-MSC migration along with TNF-α, via FAP upregulation.
